# The Role of Multimodal Generative AI in Older Adults’ Health Management: Systematic Scoping Review

**DOI:** 10.2196/84695

**Published:** 2026-05-29

**Authors:** Ting Liu, Yiming Taclis Luo, Patrick Cheong-Iao Pang, Haopeng Zhang, Ao Xiang, Qin Yang

**Affiliations:** 1 Faculty of Applied Sciences, Macao Polytechnic University, Rua de Luís Gonzaga Gomes, Macao, 999078, China, 853 8599 3815; 2 Institute of Population Research, Peking University, Beijing, Beijing, China; 3 Information Security and Assurance, Northern Arizona University, Flagstaff, AZ, United States; 4 Science in Computer Scienc, Georgia Institute of Technology, Atlanta, GA, United States

**Keywords:** GenAI, older adults, multi-modal, health management, systematic scoping review, generative artificial intelligence

## Abstract

**Background:**

The issue of population aging has emerged as a critical global challenge, driving the imperative for effective self-care and scalable health management solutions for older adults. Against the backdrop of the accelerating application of generative artificial intelligence (GenAI) in health care, a systematic evaluation is necessary to investigate how multimodal GenAI can support older adults in maintaining health and managing well-being.

**Objective:**

This study aimed to systematically evaluate the role, application contexts, empirical impacts, and developmental potential of diverse GenAI tools across critical geriatric health domains.

**Methods:**

A comprehensive search was executed across 11 major databases, including Web of Science, Scopus, PubMed, Medline, CINAHL, Cochrane, ACM Digital Library, IEEE Xplore, ScienceDirect, APA PsycInfo, and Google Scholar, with search transparency adhering to the PRISMA-S (Preferred Reporting Items for Systematic Reviews and Meta-Analyses extension for Scoping Reviews) extension.

**Results:**

A total of 28 studies met the inclusion criteria. Of the total, 82% (n=23) of the included publications were released within the last 2 years (2024‐2025). Analysis of technology revealed that over half (n=14) of the applications were based on text-driven conversational agents, while multimodal systems, leveraging generated audio, images, and sensor data, are rapidly emerging. GenAI applications were validated to support cognitive function maintenance, mental health, and chronic condition management through personalized content generation and multimodal interaction. However, current validation is primarily limited to cognitively normal, low-risk older adult populations. Persistent technical challenges include overreliance on text-based interaction, barriers in voice recognition accuracy, and suboptimal user interface adaptability.

**Conclusions:**

Preliminary evidence suggests a promising role for GenAI in enhancing older adults’ health self-management through highly personalized and multimodal interventions, particularly in cognitive and mental health support. To realize this potential and ensure equitable access, future efforts must prioritize strengthening interdisciplinary collaboration to integrate wearable technologies and edge computing, alongside establishing robust ethical frameworks to address data privacy, algorithmic bias, and the digital divide, which will be critical to building a safe, equitable, and effective environment for active aging.

## Introduction

The accelerating global aging phenomenon has emerged as one of the most pressing societal and public health challenges of the 21st century. The number of individuals aged 65 and older is projected to surge from 727 million in 2020 to 1.5 billion by 2050 [1,2]. This unprecedented demographic shift has placed immense pressure on healthcare systems, particularly in the management of multimorbidity, long-term care provision, and the allocation of increasingly scarce medical resources [3,4]. Traditional health management models, which are constrained by limited accessibility, high costs, and systemic inefficiencies, are proving inadequate to meet the complex and growing needs of the aging population [5]. In this context, leveraging emerging technologies to enhance the self-management capabilities of older adults has become a critical global priority [6].

Recent advancements in generative artificial intelligence (GenAI), particularly the emergence of core technologies, like large language models (LLMs), diffusion models, and generative adversarial networks, offer novel, scalable solutions to these pervasive geriatric health challenges [7-10]. Unlike traditional analytical artificial intelligence (AI), GenAI systems are uniquely capable of autonomously creating high-quality, multimodal content (text, images, audio, and video) [11]. This creative capacity enables GenAI applications in the care of older adults to move beyond simple data analysis, offering highly personalized, interactive, and empathetic support [12]. Initial evidence suggests GenAI is being applied across diverse domains, from LLM-based conversational systems providing preliminary diagnostic suggestions [13], to diffusion model-based technologies simplifying complex medical imaging into accessible 3D visualizations for patients [14,15]. GenAI-powered virtual companions, using emotional computing, have been shown to recognize user emotions, providing psychological support and alleviating loneliness among older adults [16,17]. The use of entertaining content generated by GenAI, such as customized storytelling or interactive games, is also reported to improve mental well-being and aid cognitive maintenance [18].

GenAI’s multimodal capability addresses the unique challenges faced by older adults. Health information presented through multimodal formats, integrating simplified text with tailored audio, images, or video, is often more accessible and comprehensible for older adult users who may experience age-related declines in vision, hearing, or manual dexterity, especially compared with traditional text-based materials [19-21]. For instance, GenAI can generate personalized health guidance tailored to an individual’s cognitive ability and lifestyle, such as creating customized diabetic care manuals that integrate textual reminders with visual exercise guides and audio cues based on real-time glucose monitoring data [22]. These diverse GenAI tools demonstrate potential in alleviating pressure on medical systems by providing 24×7 basic consultations, supporting remote monitoring for high-risk older adults, and enhancing medication adherence through natural language explanations [23,24].

Despite this profound potential, a significant gap remains in the academic literature. Existing reviews on GenAI in older adults’ health management predominantly focus on conventional, often research-based, applications like simple smartphone-based chatbots with text-only interactions [25-27]. This narrow focus neglects the rapidly expanding and critically important landscape of multimodal GenAI technologies that leverage generated visual, audio, and sensor data to overcome accessibility barriers. A comprehensive understanding of the current applications, impacts, and trends of these diverse multimodal tools across specific geriatric health domains is urgently needed to inform evidence-based policy and ethical development.

To address these limitations, this systematic scoping review adopts a rigorous PRISMA-ScR (Preferred Reporting Items for Systematic reviews and Meta-Analyses extension for Scoping Reviews) methodology to comprehensively examine the role of multimodal GenAI technologies, not limited to traditional chatbots, in older adults’ health management. This research will offer innovative and scientific evidence for policymakers, developers, and health care professionals. Specifically, this study investigates the following research questions:

1. What are the geographic and temporal trends in GenAI tool usage for older adults’ health management?
2. Which GenAI tools and multimodalities are most widely applied in this field?
3. In which specific health domains are GenAI tools used?
4. What are the roles and empirical impacts of GenAI tools in older adults’ health management?
5. What are the future potential and development trends of GenAI in this context?

## Methods

### Ethical Considerations

Ethical approval and consent to participate were not applicable as this study is a systematic scoping review that did not involve human participants or use identifiable personal data.

### Search Strategy and Protocol Transparency

This systematic scoping review was conducted following the stringent guidelines of PRISMA-ScR, with search transparency adhering strictly to the PRISMA-S (Preferred Reporting Items for Systematic Reviews and Meta-Analyses – Search extension) [28] protocol to ensure full transparency and reproducibility.

A comprehensive search was executed across 11 electronic databases: Web of Science, Scopus, PubMed, Medline, CINAHL, Cochrane, ACM Digital Library, IEEE Xplore, ScienceDirect, APA PsycInfo, and Google Scholar. The final search date was July 28, 2025.

To ensure comprehensive coverage, particularly of the multimodal focus, the search terms were meticulously refined and expanded to include concepts covering underlying models and various modalities of interaction. The search encompassed three core conceptual areas, which are (1) GenAI technology (including terms such as “Generative Artificial Intelligence,” “LLM,” “Diffusion Model,” “Image Generation,” “ChatGPT,” “Multi-modal,” and “Voice Assistant”), (2) geriatric population (“older adults” and “elderly”), and (3) health or application (“health” and “health management”). All searches were restricted to English-language articles and document types of research articles or conference proceedings. The inclusion of Google Scholar was necessary to capture cutting-edge, nonindexed, or difficult-to-locate conference papers from the computer science domain. The specific search formulas incorporating all conceptual areas for each database are summarized in Table 1. The PRISMA-ScR and PRISMA-S checklists are provided in Checklists1  2.

**Table 1.** Selected databases and search formats.

Database	Boolean search formula
Web of Science	((“generative artificial intelligence” OR “LLM” OR “Diffusion Model” OR “Image Generation” OR “ChatGPT” OR “Chatbot” OR “GenAI” OR “Multi-modal” OR “Voice Assistant”) AND (“older adults” OR “elderly”) AND (“health” OR “health management”)) AND (Document Types: Article or Proceeding Paper) AND (Languages: English)
Scopus	TITLE-ABS-KEY (“generative artificial intelligence” OR “LLM” OR “Diffusion Model” OR “Image Generation” OR “ChatGPT” OR “Chatbot” “GenAI” OR “Multi-modal” OR “Voice Assistant”)) AND (TITLE-ABS-KEY (“older adults” OR “elderly”) AND ALL (“health” OR “health management”) AND (LIMIT-TO (DOCTYPE, “ar”) OR LIMIT-TO (DOCTYPE, “cp”)) AND (LIMIT-TO (LANGUAGE, “English”))
PubMed	(((generative artificial intelligence) OR (LLM) OR (Diffusion Model) OR (Image Generation) OR (ChatGPT) OR (Chatbot) OR (GenAI) OR (Multimodal) OR (Voice Assistant)) AND (older adults OR elderly) AND (health OR health management)) Filters: Full text, English
Medline	((generative artificial intelligence) OR (LLM) OR (Diffusion Model) OR (Image Generation) OR (ChatGPT) OR (GenAI) OR (Multimodal) OR (Voice Assistant)) AND (older adults OR elderly) AND (health OR health management)
CINAHL	((MH “generative artificial intelligence”) OR TI (“LLM” OR “Image Generation” OR “ChatGPT” OR (Chatbot) OR “GenAI” OR “Multi-modal”)) AND ((MH “older adult*”) OR TI (“elderly*”)) AND ((MH “health”) OR TI (“health” OR “health management”))
Cochrane	[Title Abstract Keyword: “generative artificial intelligence”] OR [“LLM”] OR [“Diffusion Model”] OR [“Multimodal”] AND [“older adults” OR “elderly”] AND [“health management”]
ACM Digital Library	[Title: “generative artificial intelligence” OR “LLM” OR “ChatGPT” OR “Multi-modal”] AND [Title: “older adults” OR “elderly”] AND [Title: “health” OR “health management”]
IEEE Xplore	(“Full Text & Metadata”: “generative artificial intelligence” OR “LLM” OR “ChatGPT” OR “Multimodal”) AND (“Full Text & Metadata”: “older adult” OR “elderly”) AND (“Full Text & Metadata”: “health” or “health management”) Filters Applied: Conferences, Articles, Journals Filters Applied: Conferences Early Access Articles Journals
ScienceDirect	“generative artificial intelligence” AND “LLM” AND “Multimodal” AND “older adults” AND “health management” “Article type: Research articles”
APA PsycInfo	“generative artificial intelligence” AND “LLM” AND “Multimodal” AND “older adults” AND “health management”
Google Scholar	(generative artificial intelligence OR GAI OR GenAI OR AIGC OR ChatGPT OR Chatbot OR GPT OR Multimodal) AND (older adults OR elderly) AND (health OR health management)

### Data Selection and Extraction Protocol

#### Overview

All retrieved records were exported to EndNote software (Clarivate), and systematic duplicate removal was performed. The screening process was conducted in 2 sequential stages by 2 independent reviewers (TL and YTL).

#### Screening and Piloting

In total, 2 reviewers (TL and YTL) conducted the preliminary screening of article titles and abstracts based on the pre-established inclusion criteria. To ensure consistency and reliability, a piloting phase was conducted before formal screening; the 2 reviewers (TL and YTL) independently screened a random subset of 50 articles based on their titles and abstracts and calculated the preliminary κ value. This process ensured a shared and consistent understanding of the rigorous criteria.

#### Inclusion and Exclusion Criteria

The refined and strictly defined inclusion and exclusion criteria are summarized in Textbox 1. The age threshold was rigorously applied; studies were only included if the target population was 60 years and older. Furthermore, we reinforced the exclusion of studies that only explore perceptions or attitudes, prioritizing research focused on the empirical application and validated effects of GenAI technology.

Textbox 1. **Inclusion and exclusion criteria.**

**Inclusion criteria**

- Studies specifically targeting individuals aged 60 years and older.
- Research focused on generative artificial intelligence (GenAI) technologies in the field of older adults’ health care and management.
- Studies reporting the empirical application or validation of GenAI technology (quantitative, qualitative, or mixed methods).
- Publication type is research articles or conference papers.
- Full text published in English and having no time limits.

**Exclusion criteria**

- Studies primarily involving populations younger than 60 years of age.
- Research on GenAI technologies applied outside the field of older adults’ health care and management.
- Studies focusing only on older adults’ perceptions, attitudes, intentions, or opinions without actual empirical application or intervention testing.
- Review articles, theses, nonacademic publications, or book chapters.
- Full text published in other languages.

#### Quantitative Approach

Interrater reliability was calculated using Cohen κ statistic across both the screening and extraction phases. The interrater reliability was κ=0.795 for the title and abstract screening and κ=0.815 for the full-text eligibility and data extraction, both falling within the range of substantial to almost perfect agreement according to established conventional benchmarks. This agreement confirmed the reliability of the screening criteria and the robustness of the data extraction process. Discrepancies between the 2 reviewers (TL and YTL) were resolved through consensus or via consultation with a third senior reviewer (PCIP).

### Data Charting and Quality Appraisal

A detailed data extraction template was developed and refined after piloting on 5 articles. To support the multimodal and geriatric focus of this review, the extracted items were expanded to include author, year, country, research method type, specific GenAI technology and underlying model (eg, LLM), input and output content modalities (eg, text, voice, image, and sensor data), specific health application domain, target population characteristics (eg, mean age, cognitive status, level of frailty, and comorbidity), empirical impact metrics, potential, and future trends. All data were extracted by the 2 independent reviewers (TL and YTL), with a final cross-check performed for consistency.

A critical appraisal of the methodological quality of every included study was performed using the Mixed Methods Appraisal Tool (MMAT) [29]. The MMAT is a validated instrument specifically designed to assess various study designs, quantitative, qualitative, and mixed methods in systematic and scoping reviews. The assessment explicitly documented whether studies accounted for inherent GenAI model biases (eg, “hallucination”) or addressed the cultural appropriateness of their application in diverse older adult populations. The detailed MMAT scoring results were then used to inform the discussion regarding the certainty and strength of the synthesized evidence. The methodological quality of the 28 included studies [30-57] was assessed using the MMAT. Overall, the studies demonstrated moderate to high methodological quality, with strong performance in the consistency of the research question and design, as well as the clarity of data collection methods. However, the appraisal revealed deficiencies in 2 key areas, which collectively serve as crucial limitations for the current body of evidence in Table 2.

**Table 2.** MMAT^a^ quality assessment summary.

Key MMAT appraisal domain	Summary of findings	Implication for synthesis
Addressing GenAI^b^ model bias and limitations	Only 18% (n=5) of studies explicitly discussed or assessed the impact of inherent GenAI model biases (eg, “hallucination” or training data bias) on their results.	Reliability of findings: the lack of attention to bias means the safety and trustworthiness of these GenAI applications remain uncertain, requiring cautious interpretation of their reported effects.
Cultural and ethnic appropriateness of application	Fewer than 11% (n=3) of studies explicitly designed cross-cultural evaluations or validated the suitability of GenAI tools across diverse ethnic or cultural older adult populations.	Generalizability of findings: results are highly concentrated in specific cultural or regional contexts (eg, China and United States), severely limiting the universality and equity of the findings across the global aging population with varying health care systems and values.
Compliance, intervention fidelity, and loss to follow-up	Most studies provided clear data on intervention compliance, but several quantitative studies lacked robust reporting on the handling of attrition rates in long-term follow-up.	Long-term efficacy: the lack of long-term validation and detailed attrition analysis restricts the ability to evaluate the sustained effectiveness of GenAI in older adults’ health management.

a MMAT: Mixed Methods Appraisal Tool.

b GenAI: generative artificial intelligence.

### Collating, Summarizing, and Reporting the Results

The findings were systematically collected, summarized, and analyzed using descriptive statistics to characterize the trends and features of the included articles (eg, temporal growth, technology distribution, and geographical concentration). Subsequently, a narrative synthesis was performed using these descriptive findings to rigorously address the research questions raised in the review. This process involved synthesizing the empirical impact reported by the studies, identifying recurring themes in application and challenge, and mapping the evidence across different geriatric health domains. All authors verified the final synthesis and interpretation.

### Quality and Risk of Bias Assessment

According to Tricco and colleagues [58], and considering the peculiarity of the scoping review, we did not appraise the methodological quality or risk of bias of the included papers.

## Results

### Overview

**Figure 1.**
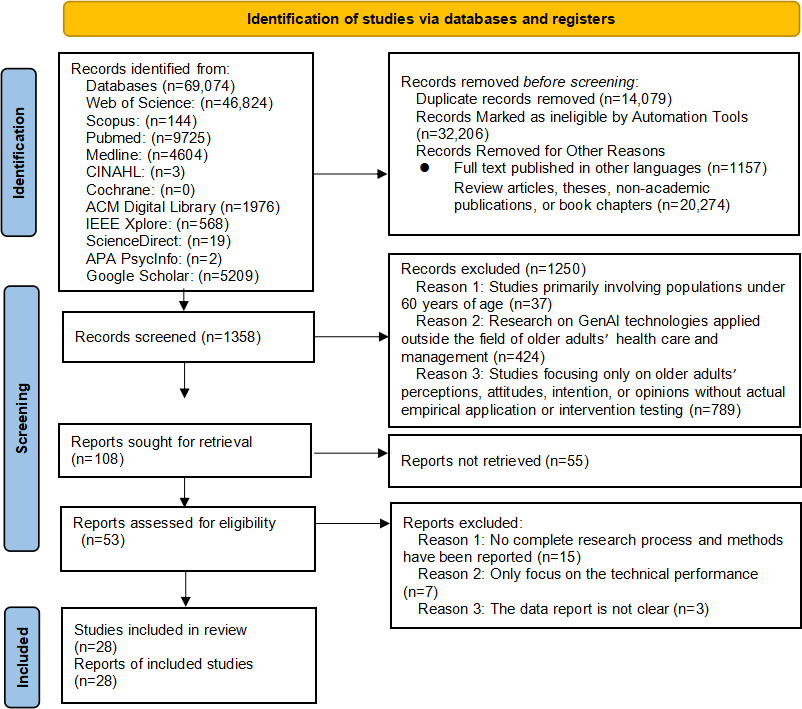
PRISMA (Preferred Reporting Items for Systematic Reviews and Meta-Analyses) flowchart.

The systematic search across the 11 electronic databases initially yielded a total of 69,074 records. The selection process, detailed in the PRISMA (Preferred Reporting Items for Systematic Reviews and Meta-Analyses) flow diagram (Figure 1), began with a thorough cleaning stage where 14,147 duplicate records were removed. An additional 32,206 records were marked as ineligible by automation tools, and 22,721 records were removed for other nonacademic reasons, specifically including 1354 articles published in other languages and 21,367 classified as review articles, theses, nonacademic publications, or book chapters. The title and abstract screening then commenced with 1358 unique records. This phase resulted in the exclusion of 1250 records, with the main reasons being studies focusing on populations younger than 60 years of age (n=37), research outside the field of older adults’ health care (n=424), and studies focusing only on older adults’ perceptions or attitudes without actual empirical application (n=863). This rigorous screening left 108 reports sought for full-text retrieval. Of these, 55 reports could not be retrieved, leaving 53 reports assessed for eligibility. The full-text assessment resulted in the final exclusion of 25 reports due to the lack of a complete research process and methods (n=15), a sole focus on technical performance (n=7), or unclear data reporting (n=3). Ultimately, a final set of 28 articles [30-57] met all inclusion criteria and were included in the scope of this systematic review (refer to Table 3 for article characteristics).

**Table 3.** Overview of study characteristics.

Author (year, country)	Research methods	GenAI^a^ technology	Input and output content type	Health application	Target population	Significance	Impact	Potential and future trends
Satoh et al (2024, Japan) [30]	Mixed (Shapiro-Wilk test, Wilcoxon signed-rank test, comments of online meeting)	Music Trinity Generative Algorithm‐Human Refined (MusicTGA-HR^b^)	Text and music audio	Neurology-frontal lobe functions and memory	118 cognitively normal older adults	MusicTGA-HR intervention improves frontal lobe functions and memory in older adults	Generating music through text can help older adults without professional music training to conduct non-pharmaceutical cognitive training, thereby enhancing their cognitive abilities	The use of GenAI can replace face-to-face nonpharmaceutical cognitive training activities. More professionals will be needed to assist older adults
Pramod et al (2024, India) [31]	Quantitative (partial least squares structural equation modeling)	GenAI system	—^c^	Older adult health management	314 older adults	GenAI provides positive emotional support for the older adults	GenAI benefits personalized older adult care via perceived ease of use	GenAI tools should have a user interface, features, and functional design that are more suitable for the older adults
Xie et al (2024, China) [32]	Qualitative (observation and in-depth interviews)	AI^d^ chatbot	Text	Older adult health management-exercise	6 older adults	AI chatbots help the older adults access professional exercise information effectively	AI chatbot exercise partner and community support, transforming the older adult’s exercise perceptions	Improve the AI chatbot by leveraging more advanced language models and optimized prompts to mitigate the risk of generating
Yu et al (2024, China) [33]	Quantitative (pre-post controlled study independent *t* tests)	Horticultural therapy combined with AIGC^e^	Artworks	Mental health–older adult depression	20 older adults with depression	AIGC-horticultural therapy reduces depressive symptoms and enhances mental health in older adults	AIGC-horticultural therapy could be an effective intervention to reduce depressive symptoms	Explore the mechanisms and long-term effects in depth
Jin et al (2024, China) [34]	Qualitative (thematic analysis)	GenAI music video	Text, image, video, and music audio	Memory	10 older adults	GenAI combined with music reminiscence improves memory in older adults	Language, tone, and emotions affect willingness to interact with GenAI; some perceive AI as lacking emotional warmth	GenAI requires more emotional expression to evoke emotional resonance, and it also demands higher-quality and more accurate image and content output
Yun et al (2024, Korea) [35]	Mixed (controlled study paired *t* tests, and poststudy interviews, thematically analyzed)	AI chatbot	Text, and text-based games	Neurology-dementia	123 older adults	AI chatbots based on AI demonstrate effectiveness in early dementia screening and personalized cognitive training	GenAI enhances cognitive and emotional well-being in older adults	AI chatbot designs should incorporate personalized and emotionally intelligent interactions. Include positive reinforcement, tailored emotional responses, and interactive elements, and integrate social connectivity
Dragut et al (2025, Spain) [36]	Quantitative (evaluation of tests)	AI chatbot	Text, voice, and video	Language pathology-speech disorders	20 older adults	GenAI conversational agents based on LLM^f^ optimize language support and interaction for the elderly	GenAI dialog agents correct language, alleviating comprehension limitations	GAI agents are currently unable to handle user voices that exceed a certain sound threshold. The future will need to be trained on data with different speech disorders or specific speech features
Khan et al (2024, Pakistan) [37]	Quantitative (evaluation of tests)	GenAI system for the older adult–assisted living situations	Physiological parameters, environmental information, and text	Older adult health management	30 older adult patients	GenAI systems enhance user-system interaction and feedback mechanisms	GenAI systems provide timely, personalized health information, improving satisfaction, and reducing caregiver burden	Enhanced training programs, advanced privacy techniques, collaborative integration, scalable infrastructure, cost-effective solutions, interoperability standards, autonomous learning algorithms, ethical AI frameworks, and user-centric design
McCarren et al (2025, Sweden) [38]	Mixed (semistructured interviews and questionnaire)	AI chatbot	Text	Mental health-mindfulness	15 older adults	LLMs create personalized AI mindfulness coaches for older adults	Voice interaction, responsiveness, and ease of use enhance the GenAI experience	Enhance interactivity and personalized guidance
Tsai et al (2021, Taiwan) [39]	Quantitative (Cronbach α value)	AI chatbot	Text	Older adult health management-health knowledge learning	14 older adults	AI chatbots help the older adult quickly understand and access health information	Stable systems with minimal errors build user trust; users seek disease prevention knowledge via chatbots	The content is mainly provided with pictures, texts, and videos. The pictures and texts can accelerate the understanding of disease prevention in a short time, and the videos can clearly explain the content information
Chou et al (2024, Taiwan) [40]	Quantitative (the Fisher exact test, the Wilcoxon signed-rank test, analysis of covariance, and Spearman rank correlation)	AI chatbot	Text, image, and voice	Mental health-stress	35 older adults	AI chatbots improve health information accessibility, awareness, and quality of life	Chatbots benefit psychiatric outpatients with stable depression or anxiety, reducing loneliness	Explore how more personalized mental health support can be provided by expanding on the functionality of the chatbot to effectively manage depression and anxiety among older adults
Bennion et al (2020, United Kingdom) [41]	Mixed (qualitative description, and mixed 2×3 analysis of variance, post hoc 2-tailed *t* tests, independent *t* tests, and simple linear regression)	AI chatbot	Text	Mental health	112 older adults	AI chatbots alleviate loneliness and self-disclosure difficulties in the older adults	AI chatbot has the potential for development in the field of electronic dialogue-based psychological therapy for the older adults	Further evaluate the clinical and health economic utility of conversational agents, context needs to be more clinical, outcomes need to be assessed over longer periods, and system usability needs
Reed et al (2025, United States) [42]	Mixed (interviews—thematic analysis, nonparametric test, and Wilcoxon signed-rank test)	GenAI images	Text and image	Memory	34 older adults with at least 1 major chronic illness, and English literacy (spoken)	Personalized GenAI image interventions maximize mental health benefits for long-term care residents	GenAI-generated images from memories facilitate reminiscence, positive emotions, and social interaction in long-term care	Need a more diverse sample and how it affects mood for patients with a lower depressed mood at baseline. More randomized controlled trials are needed to evaluate the efficacy of mindfulness-based art therapies. whether GenAI images are superior to reflection, discussion, and empathy alone
Bosco et al (2024, United States) [43]	Qualitative (thematic analysis)	Voice assistant, and AI chatbot	Text and voice	Neurology—Alzheimer disease and related dementias	15 older African American and Black adults	GenAI requires accessible content and technology to build trust and meet interaction preferences	Historical medical mistrust leads to reliance on traditional media (eg, television) for health information	Need to design a GenAI technology of credible and accessible content, fosters users’ representation and control, a time-efficient, and leverages preexisting community processes
Naseer et al (2025, Saudi Arabia) [44]	Quantitative (evaluation of tests)	Adaptive AI	Fall data and alerts	Older adult health management-Fall	198 fall events	GAN^g^ and IoT^h^ sensors monitor health indicators, medication adherence, and environmental activities	The positive impact of the discussed AI system in enhancing care efficiency and the older adult people’s rights and safety	New enhancements are possible through wearable devices powered by edge AI, including edge processing for on-the-spot decisions and without delays inherent in cloud processing, and vital scalability regarding seamless interoperability between the differing devices.
Kim et al (2024, Korea) [45]	Quantitative (multiple regression analysis, paired sample *t* test analysis)	AI chatbot	Text, image, and game	Memory	32 older adults	Chatbots foster therapeutic relationships and meaningful connections with the older adults	Increased adherence to the AI chatbot was correlated with reduced depression. The participants often shared sentiments reflecting a deeper connection with the chatbot	Explore the variable impacts of personalized cognitive training across different cognitive domains
Bosco et al (2025, United States) [46]	Qualitative (think aloud—thematic analysis)	Voice assistant, and AI chatbot	Text and voice	Neurology—Alzheimer disease and related dementias	15 Black American older adults	Poor GenAI design may increase risks for marginalized communities	Usability, accessibility, cultural relevance, and adoption are important for GenAI tools. Consent, privacy, and bias can impact the use of GenAI for an underrepresented community	Create an inclusive and culturally relevant AI-supported platform for health will follow
Olszewski et al (2024, Poland) [47]	Quantitative (Shapiro-Wilk test)	AI chatbot	Text	Older adult health management	303 older adults and 270 young adult students	Most older adults prefer face-to-face interactions over chatbots	Older adults prefer traditional media (eg, television) despite pandemic-era tech advances	Greater involvement of older adults in activities involving the use of technology-smartphones, computers, and software
Liang et al (2025, Hong Kong) [48]	Quantitative (Markov chain Monte Carlo, chi-square, and independent sample *t* tests)	AI chatbot	Text	Older adult health management—physical activity	194 older adults	Chatbots improve compliance with web-based health interventions	With evolving knowledge graphs and machine learning, the chatbot can ensure the accuracy of human-machine interactions, which further increases its efficacy	Need a bigger data size and a longer follow-up period of the intervention
Miura et al (2022, Japan) [49]	Mixed (interview and questionnaire)	AI chatbot	Text, voice, and image	Older adult health management—physical activity	8 older adults and 19 younger adults	AI chatbots facilitate self-reflection on health conditions	The individuality of its effects still needs to be deeply studied as a case study, including personal culture, habits, and life rhythms	Linking a chatbot and a wearable smartwatch to achieve real-time health tracking and timely personalized health care questionnaires
Wang et al (2021, United States) [50]	Qualitative (evaluation of tests)	AI chatbot	Text	Older adult health management	1690 questions	AI chatbots deliver real-time, personalized, and accessible health information without technical barriers	Chatbots offer natural interfaces without technical background requirements, but this may hinder technology adoption	Plan to deploy the AI chatbot voice assistant in a real setting
Thetbanthad et al (2025, Thailand) [51]	Quantitative (evaluation of tests)	Visual question answering model-based LLM	Text and image	Older adult health management	100 prescription labels	Zero-shot AI models clarify medication labels for better older adult medication management	Integrated models provide accessible, accurate, personalized drug information for older adult patients or providers	The effectiveness of AI-driven systems for enhancing drug label interpretation and medication management
Khamaj (2025, Saudi Arabia) [52]	Quantitative (focus groups and semistructured interviews)	AI chatbot	Text	Older adult health management	—	AI-enhanced chatbot interfaces address usability challenges in medical technology for older adults	Tiered functionality based on technological proficiency enhances accessibility, improving medical management and outcomes	More diverse elderly participants can be recruited to obtain more comprehensive data and insights. Ensure the availability and consistency of the Chatbot across different devices. Collaborate with healthcare providers and industry experts. Continue to implement strong security measures to protect users’ personal health information. Through continuous iterative development and usability testing. Encourage users to consult medical professionals while following the guidance provided by the chatbot to ensure safe and reliable decisions
Imkome (2025, Thailand) [53]	Quantitative (means, frequencies, and SDs)	AI chatbot	Text	Mental health	44 older adults	AI chatbots support mental health improvement in older adults	Brief GenAI interactions yield meaningful benefits	Should refine ethical frameworks to ensure the responsible implementation of these technologies while assessing their effectiveness and ethicality. To address intrasubject variability, one should use repeated measures for a more robust estimation of the intervention’s impact. Exploring multimodal methodologies, such as text, voice, and video, to tailor interventions based on individual user data is necessary. Integrating feedback systems is crucial for continuously enhancing the user experience
Piau et al (2019, France) [54]	Quantitative (mean values and SDs)	AI chatbot	Text	Cancer	9 older adults	AI chatbots reduce nurses’ workload, enhance symptom monitoring, and improve quality of life management of patients with cancer, and the quality of life of older adult patients with cancer	It helps optimize the home follow-up of patients undergoing chemotherapy. Even for the older adult patient group with advanced age and limited technical literacy, they can also accept and use the health monitoring technology based on GenAI	Expand the sample size and follow-up period to assess the long-term effects. Develop simpler usage methods for patients who live alone or have low technological literacy. Further optimize the system functions. Adding auxiliary functions like voice interaction, conducting randomized controlled trials to verify its clinical effectiveness. Continuously evaluate the implementation process and usage experience of the technology in the actual medical environment
Lin et al (2025, China) [55]	Quantitative (evaluation of tests)	EHMQA^i^-GPT^j^	Text	Elderly health management	26 million question-answer pairs	LLM-based EHMQA-GPT integrates knowledge related to older adult health management through text data research	Investigate the cross-lingual scalability of instruction-tuning and evaluation pipelines, leveraging multilingual instruction datasets and alignment strategies to assess the generalizability of GPT in multilingual, multicultural, and low-resource settings	GPT framework to broader health domains, by expanding the corpus to cover diverse subfields of medicine
Wang et al (2025, Hong Kong) [56]	Quantitative (randomized clinical trial)	AI chatbot	Text video	PV^k^ uptake	374 participants, mean age 69.6 (SD)	The chatbot-delivered interventions addressed some main modifiable barriers to uptake of PV among older adults, including lack of awareness about benefits and low self-efficacy, and concerns about adverse effects, high cost, and inconvenience related to PV	These vaccine chatbots required some digital health literacy to operate, which could be challenging for individuals of low socioeconomic status. Most vaccine chatbots are operated via smartphones, which presents challenges of accessibility	Few vaccine chatbots have been evaluated in low- and middle-income-countries. Future studies should explore the feasibility and effectiveness of vaccine chatbots in these countries
Wang and Li (2024, China) [57]	Mixed (interview and questionnaire)	AI chatbot	Text, voice	Emotional health	15 participants	In the short term, both ChatGPT companionship and mindfulness meditation had rather limited impacts on significantly reducing depression symptoms and feelings of loneliness in older persons based on trials conducted with older adult patients in nursing homes	In contrast to conventional mindfulness therapies, cutting-edge AI-based interactive modes like ChatGPT might also be useful for reducing negative emotions and enhancing the mental health of older adults	In the future, offering older adults a variety of easy social and psychological support channels through the use of technology and psychological interventions would be crucial

a GenAI: generative artificial intelligence.

b MusicTGA-HR: Music Trinity Generative Algorithm‐Human Refined.

c Not applicable.

d AI: artificial intelligence.

e AIGC: artificial intelligence–generated content.

f LLM: large language model.

g GAN: generative adversarial network.

h IoT: internet of things.

i EHMQA: Elderly Health Management Questions and Answers

j GPT: Generative Pre-trained Transformer.

k PV: pneumococcal vaccination.

### Characteristics of Studies

**Figure 2.**
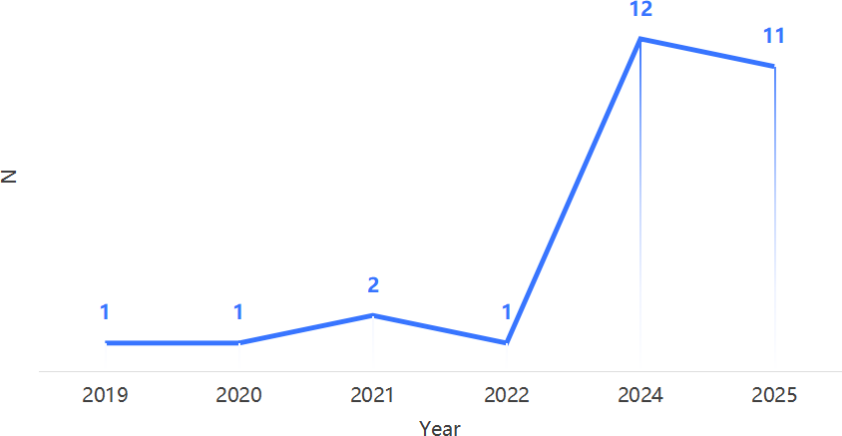
Annual number of publications.

The final analysis incorporated 28 studies [30-57], with trends in publication and regional distribution characteristics of GenAI research in older adults’ health examined (Figures2  3). Temporally, the studies exhibit a clear exponential growth trajectory—the annual publication rate averaged only 1.25 articles (SD 0.50) from 2019 to 2022, which then surged to 12 in 2024 and 11 in 2025 (overall mean 4.67, SD 5.32). This recent output accounts for more than 82% (23/28) of the total publications, indicating that GenAI applications are in a phase of rapid emergence driven by breakthroughs in GenAI technology, the escalating global aging crisis, and strategic policy emphases on digital health.

**Figure 3.**
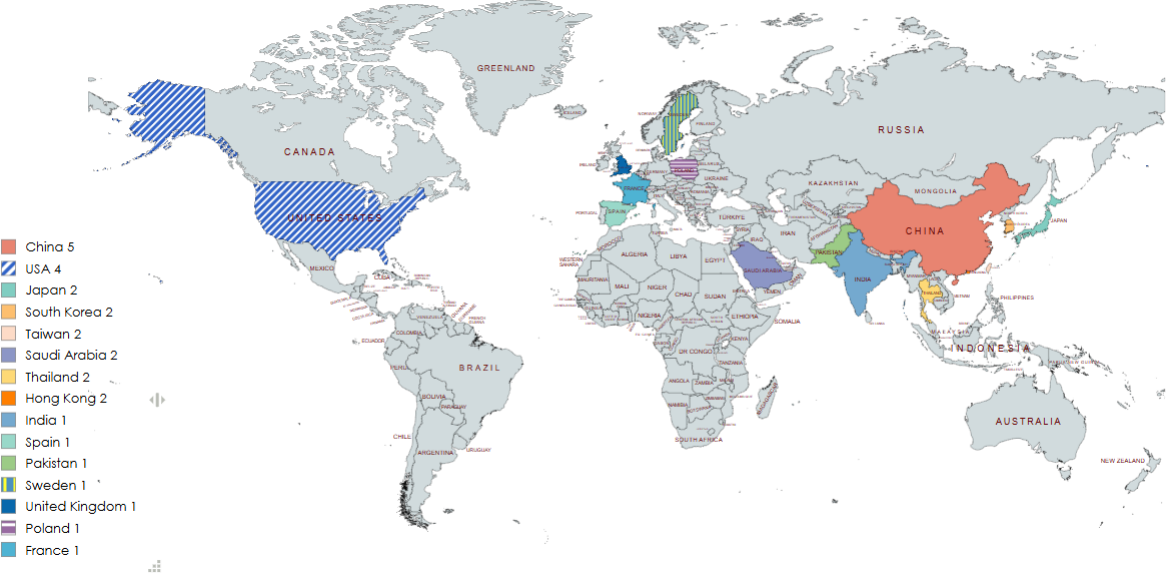
Countries of publication.

Geographically, research output demonstrates significant regional concentration. The United States (n=4) [42,43,46,50] and the China region (China: n=5 [32-34,55,57] and Hong Kong: n=2 [48,56]) collectively contributed 11 studies (11/28, 39.2%), establishing a clear leading position aligned with their synergistic advantages in cutting-edge research and development and vast older adult market demand. Following these leaders are countries or regions with specific aging pressures or policy interests, including Japan (n=2) [30,49], South Korea (n=2) [35,45], Taiwan (n=2) [39,40], Saudi Arabia (n=2) [44,52], and Thailand (n=2) [51,53]. European welfare states and developing nations also contribute to the literature, highlighting the diverse, context-specific demands for GenAI in addressing global aging challenges.

Methodologically, the studies predominantly used quantitative approaches (n=16) [31,33,36,37,39,40,44,45,47,51-57], serving as the primary evaluation framework to validate tool efficacy through statistical techniques like pre-post control designs and correlation tests. These were complemented by mixed methods research (n=7), which integrated quantitative tests with qualitative interview techniques for comprehensive data cross-validation, and qualitative studies (n=5) [32,34,43,46,50], which prioritized in-depth exploration of user subjective experiences, perceived barriers, and needs through thematic analysis and observational protocols (Table 4).

**Table 4.** Research method types of studies.

Research method type	Frequency, n	Specific methods
Quantitative	16	Evaluation of tests; pre- and postcontrolled study, independent *t* tests; Cronbach α value; multiple regression analysis; paired-sample *t* test analysis; Shapiro-Wilk test; Markov chain Monte Carlo; chi-square test; independent sample *t* tests; the Fisher exact test; The Wilcoxon signed-rank test; Spearman rank correlation; means, frequencies, and SDs; mean values and SDs
Qualitative	5	Thematic analysis; observation and in-depth interviews; think-aloud thematic analysis; evaluation of tests
Mixed	7	Controlled study-paired *t* tests and poststudy interviews-thematically analyzed; semistructured interviews and questionnaire; mixed ANOVA, post hoc 2-tailed *t* tests, independent *t* tests, simple linear regression; interviews—thematic analysis; nonparametric test; Wilcoxon signed-rank test; qualitative description; mixed analysis of variance; post hoc 2-tailed *t* tests; independent *t* tests; simple linear regression; interview and questionnaire

### Types of GenAI Technology

Regarding technological type (Table 5), general GenAI systems (basic chatbots; n=13) remain the most frequent single category, serving as the foundational technology for health information interaction and simple task automation. These basic systems are complemented by vertically integrated applications, which expand technical boundaries through GenAI+multimodal/specific application (n=14), encompassing systems for assisted living, multimedia generation, and therapeutic integration. The core value of GenAI technologies lies in multimodal interaction support and personalized service generation (n=3), where studies have integrated voice assistants with chatbots and leveraged visual question-answering models to reduce operational barriers and enable more natural conversational flows. However, the current technological landscape exhibits a homogenization tendency, with over one-third of the studies concentrating on basic health consultations, underscoring the need for future research to broaden GenAI’s functional boundaries in older adults’ health, validating cutting-edge directions, such as affective computing and ethical risk control.

**Table 5.** Types of GenAI^a^ technology.

GenAI technology type	Frequency, n	Core functions or application scenarios
General GenAI systems (basic chatbots)	13	Basic health information interaction, chronic disease management support, and simple task automation serve as the foundational technology for other derivative applications
GenAI+personalization (specialized LLMs^b^ or chatbots)	3	Dynamic generation of customized health solutions, adjustment of companionship strategies based on mood analysis, and improving service precision through user data adaptation
GenAI-generated multimedia (music video or image)	3	Generation of personalized health education content, psychological rehabilitation support materials, enhancing information comprehensibility and engagement through multimodal formats
GenAI for assisted living	2	Elderly home-based health monitoring, environment perception, and demand prediction; age-friendly interaction design tailored for home settings
GenAI+multimodal interaction	4	GenAI chatbots integrated with voice assistants, visually question-answering model-driven image-text interaction for health consultations, reducing operational barriers, and optimizing natural interaction
Advanced GenAI applications	2	Adaptive AI^c^ systems, exploratory applications for complex health decision support; currently low in proportion but indicating potential for deep technical integration
GenAI in therapy integration	1	Integration of generative content with horticultural therapy to assist in older adult psychological intervention and emotional management.

a GenAI: generative artificial intelligence.

b LLM: large language model.

c AI: artificial intelligence.

### Types of Input and Output Data

The data interaction patterns demonstrate a clear multimodal input-output integration trend, built upon a text-based foundation (Table 6). Text (n=6) remains the most universally accessible interaction medium, but its relatively low frequency suggests that GenAI applications heavily rely on multimodal combinations to meet the diverse needs of older users. This is primarily due to the text’s low technical barrier and alignment with older adults’ communication preferences. Beyond text, studies integrate alternative input modalities such as voice (n=8), images (n=10), and physiological or environmental parameters (n=2) to enhance monitoring accuracy and address complex health issues. For output, text is the primary feedback modality, yet multimodal outputs serve as crucial supplements: music or audio (n=2) for mood regulation, images or videos (n=5) for visualizing complex health information, and gamified text (n=2) for cognitive training. Although multimodal applications are concentrated in specific scenarios, the overall model is text-centric, supplemented by multimodal elements (eg, images, voice, and physiological data) to meet older users’ needs for visual preferences, operational ease, and emotional support.

**Table 6.** Types of input and output data.

Data type (input or output)	Frequency, n	Core functions or application scenarios
Text	6	Basic health consultations, delivering health advice, explaining diseases, and providing emotional support; the most universally accessible interaction medium.
Text+voice	4	Voice-based interactions and feedback, reducing operational difficulty, and enhancing naturalness and accessibility.
Text+image	5	User-uploaded images for auxiliary diagnosis, generating visualized health information; image output lowers comprehension barriers.
Text+voice+image	4	Multichannel interactions, combining textual logic, vocal naturalness, and visual intuitiveness for comprehensive health support.
Text+music audio	2	Emotional regulation, rehabilitation assistance; targets mental health needs and movement rehabilitation.
Text+video	2	Dynamic health education and virtual companionship content, enhancing the engagement and dissemination effectiveness of health knowledge.
Physiological parameters+environmental info+text	1	Acute risk warning and environmental adaptation; input of physiological parameters and environmental data supports real-time health risk identification and context-aware interventions.
Text+game	1	Gamified cognitive training interactions to improve older adult engagement and cognitive exercise effectiveness.
Artworks	1	Generation of art therapy content as a supplementary tool for psychological interventions.
Fall data+alerts	1	Fall monitoring and emergency response; dedicated to acute prevention scenarios.
Text+image+game	1	Integrated cognitive training and entertainment; multimodal intervention design targeting maintenance of elderly cognitive functions.

### Types of Health Application

The applications of GenAI primarily concentrate on 4 core domains—neurocognitive health, mental health, comprehensive health management, and specific disease interventions (Table 7). Comprehensive health management (n=12) covers a broad foundational range, including health knowledge acquisition, physical activity, and fall prevention. These applications use GenAI primarily for information dissemination and behavioral reminders. Neurocognitive-related health issues (n=8) dominate the landscape, encompassing subdomains, such as Alzheimer disease and related dementias, memory, and speech disorders. These applications leverage GenAI’s multimodal and personalized capabilities to address age-associated cognitive decline through tailored interventions, emphasizing early prevention and functional maintenance. Mental health support (n=6) represents another high-frequency area, spanning depression, stress, and general well-being. GenAI excels here by simulating empathetic dialogue (affective computing) and delivering standardized psychological protocols via multimodal formats, often forming a “cognitive-emotional” synergistic intervention model. Specific disease interventions (n=2; eg, management of patients with cancer and medication management) remain rare, likely due to the complexity of disease mechanisms and stringent data privacy requirements. This distribution reflects a strategic focus on addressing urgent neurocognitive and mental health needs while promoting broad-based health maintenance.

**Table 7.** Types of health application.

Data type (input or output)	Frequency, n
Comprehensive health management	12
Neurocognitive health	8
Mental health	6
Specific disease interventions	2

### Target Population

The research primarily targets 3 key cohorts. First, older adults with normal cognitive function constitute the main subjects for evaluating GenAI’s generalizability in basic health management and technology acceptance testing. Second, chronic disease populations and older adult patients form a critical cohort, focusing on GenAI’s role in addressing complex health demands like chronic disease management, postoperative rehabilitation, or acute condition prevention, which necessitates enhanced personalization and multimodal data integration. Third, mental health-focused populations are key subjects in specialized demand scenarios, assessing GenAI’s efficacy in emotional support and psychological intervention. The gradual inclusion of ethnic minority groups and populations with unique social attributes (eg, African American and Black adults) reflects growing attention to health equity, aiming to investigate GenAI’s adaptability and cultural relevance in diverse contexts. A small subset of studies also targets special health status populations, such as individuals with borderline cognitive impairment or those facing significant barriers to technology adoption.

## Discussion

### Main Findings and Results of Studies

This study, based on a systematic analysis of 28 articles [30-57], reveals the core characteristics and findings of GenAI applications in older adults’ health. Temporally, the research exhibits a growth trend over the past 2 years, reflecting intensifying global aging challenges and the rising maturity of GenAI technology. Geographically, research output is concentrated, with the United States and China leading the field by leveraging their advanced AI foundations and proactive smart older adults’ care policies. Methodologically, quantitative research is the dominant approach, supplemented by qualitative and mixed methods designs. The technology focus is on general GenAI systems, which form the core, expanding to specialized multimodal content generation and integrated applications complemented by interaction tools like voice assistants and visual question-answering models. Multimodal inputs (voice, images, and physiological parameters) and outputs (music, video, and gamified text) are increasingly used for personalized health services, demonstrating a technological shift from single-text to composite interactions. Health applications primarily cover neurocognitive health, mental health, and comprehensive health management, with the target populations ranging from ordinary older adults with normal cognition to those with chronic diseases and specific mental health concerns. The inclusion of ethnic minorities reflects growing attention to health equity and marginalized group needs.

### Significance and Role of GenAI for Older Adults’ Health

GenAI demonstrates particular significance across 3 functional dimensions—cognitive preservation, mental well-being, and health management efficiency. In the cognitive domain, interventions like MusicTGA-HR and GenAI-based image recall therapy effectively improve frontal lobe function and memory performance, validating GenAI’s potential in maintaining cognitive reserve and delaying decline through personalized adaptation [30,43,46]. For mental health support, GenAI, through affective computing and empathetic dialogue, serves as a critical tool for alleviating loneliness and psychological stress, with general-purpose AI chatbots fostering self-disclosure and simulating the emotional warmth of human companionship [39,57]. Complementary AI-generated content, such as horticultural therapy integration, further expands psychological intervention scenarios, significantly reducing depressive symptoms [33,59]. Furthermore, in health management, LLM-based chatbots enhance accessibility by transforming complex medical information into simple, intuitive content, while integrating Internet of Things (IoT) sensor data for real-time tracking of critical health indicators and personalized interventions in chronic disease management [37,43,46]. Collectively, these applications establish a robust support system, advancing health equity by offering high-quality services to underserved groups and optimizing health care resource allocation by automating information delivery and monitoring [60].

### Impact, Benefits, and Challenges of GenAI for Older Adults’ Health

The application of GenAI yields significant benefits in maintaining cognitive function, supporting mental health, and promoting health management autonomy, alongside facing adoption challenges [7]. Key benefits include personalized cognitive training (eg, text-to-music interventions) and psychological intervention via empathetic dialogue, which alleviates loneliness and fosters long-term self-reflection, leading to a “cognitive activation effect” [30,34,42,61]. GenAI also enhances self-efficacy by improving information accessibility and operational convenience, simplifying complex medical knowledge, and using real-time IoT data for timely health alerts [44,54]. GenAI also enhances self-efficacy by improving information accessibility and operational convenience, simplifying complex medical knowledge, and using real-time IoT data for timely health alerts [62,63]. Moreover, suboptimal system responsiveness and a lack of affective computing modules often trigger perceptions of machine coldness, leading to doubts about the reliability of AI-generated content [47,64]. Digital access barriers (small fonts and complex workflows) and concerns over privacy, algorithmic bias, and potential errors further deter adoption [65,66]. GenAI’s long-term potential requires robust policy and ethical frameworks, as well as advancements in voice interaction, multimodal fusion, and adaptive learning algorithms to overcome interaction bottlenecks and ensure service equity [36,67].

### Potential and Future Trends of GenAI for Older Adults’ Health

Future development trends for GenAI in older adults’ health will center on 4 key dimensions—technological deepening, interaction optimization, population adaptation, and ethical safeguards. Technologically, research must delve into the long-term effects and mechanisms of GenAI psychological interventions, expanding chatbot functionalities to integrate therapies like personalized art and mindfulness-based feedback for targeted emotional management [33]. Interaction optimization will drive the evolution from current text-based tools toward immersive multimodal experiences, combining voice, imagery, haptics, and olfaction, with designs prioritizing older adults’ physiological and psychological traits (eg, large-font interfaces and voice-input alternatives) to reduce cognitive load [68]. In terms of population adaptation, widespread adoption requires professional guidance, including training caregivers in digital literacy, and developing tiered tool systems (eg, basic 1-click operation for novices) to enhance accessibility for low technology-proficiency groups [69]. Ethically, advancements like edge-AI–powered wearables will enhance privacy by enabling localized data processing [44]. Crucially, transparent ethical frameworks must prioritize data privacy protection, implement user-controlled mechanisms, and use cross-disciplinary ethics review boards to assess risks like algorithmic bias and emotional dependency, ensuring technological innovation upholds dignity and rights [70,71].

### Limitations

This study, based on a systematic analysis of 28 articles [30-57] focused on GenAI applications in older adults’ health, presents several limitations that may affect the generalizability and external validity of the conclusions. First, the included studies exhibit insufficient sample representativeness. The research populations predominantly consisted of older adults with normal cognitive function, while studies targeting high-risk groups, such as those with cognitive impairments, multiple chronic disease comorbidities, or advanced age, were scarce. Furthermore, marginalized populations were critically underrepresented, meaning the conclusions primarily reflect the needs and experiences of “technology-adaptive” subgroups rather than the full diversity of the global older adult population. Second, the intentional exclusion of non–application-focused studies, such as reviews, nonacademic publications, and research emphasizing only user attitudes, perceptions, or intentions toward GenAI, limits the comprehensiveness of the review. Although this exclusion maintained a focus on practical implementation, it potentially reduced insights into the broader contexts that significantly shape GenAI adoption and effectiveness. These limitations necessitate cautious interpretation of the findings. Future research must prioritize expanding sample diversity to include high-risk and marginalized populations, enhancing long-term follow-up designs, optimizing real-world application testing, and deepening ethical and mechanistic explorations to advance the safe, effective, and equitable application of GenAI in older adults’ health.

### Conclusion

This systematic scoping review analyzed 28 studies sourced from 11 electronic databases, summarizing the efficacy, current application status, and future potential of multimodal GenAI in promoting older adults’ health. The findings confirm that GenAI offers benefits across the care spectrum, particularly in maintaining cognitive function, supporting mental health, and enhancing comprehensive health management. Its efficacy is fundamentally driven by its core strengths—multimodal interaction capabilities and the ability to provide highly personalized content. However, most interventions are short-term, lacking robust long-term efficacy data and detailed mechanistic exploration. Studies overwhelmingly focus on low-risk, cognitively intact older adults, leaving a critical gap in coverage for high-risk and marginalized populations. Furthermore, persistent challenges related to age-friendly interaction design and unresolved ethical risks continue to hinder equitable adoption. Future research must prioritize inclusivity by expanding investigations into high-risk and underserved populations, optimizing multimodal interaction designs and age-friendly interfaces, and establishing long-term follow-up studies to validate long-term effects.

## Supplementary material

10.2196/84695 Checklist 1 PRISMA-ScR checklist.

10.2196/84695 Checklist 2 PRISMA-S checklist.

## Abbreviations

AI: artificial intelligence
GenAI: generative artificial intelligence
IoT: Internet of Things
LLM: large language model
MMAT: Mixed Methods Appraisal Tool
PRISMA: Preferred Reporting Items for Systematic Reviews and Meta-Analyses
PRISMA-S: Preferred Reporting Items for Systematic Reviews and Meta-Analyses – Search extension
PRISMA-ScR: Preferred Reporting Items for Systematic reviews and Meta-Analyses extension for Scoping Reviews

## References

[1] Crampton A (2009). Global aging: emerging challenges. https://www.bu.edu/pardee/files/2009/09/pardee_aging-6-global-aging.pdf.

[2] Rabadi SA (2023). The world demographically is similar to Europe: problematic and challenges. https://www.researchgate.net/publication/378486783_The_World_Demographically_Is_Similar_To_Europe_Problematic_and_Challenges.

[3] (2024). Demographic shifts and healthcare: a review of aging populations and systemic challenges. Int J Sci Res Arch.

[4] Mayhew LD (2000). Health and elderly care expenditure in an aging world. https://pure.iiasa.ac.at/id/eprint/6109/1/RR-00-021.pdf.

[5] Khan HTA, Addo KM, Findlay H (2024). Public health challenges and responses to the growing ageing populations. Public Health Chall.

[6] Liu T, Luo YT, Pang PCI (2025). Digital technologies-enhanced older adults health management: developing a five-dimensional extension of social learning theory for community settings. Front Public Health.

[7] Sankaran A, Singla MP (2024). Artificial intelligence in geriatric healthcare: opportunities and challenges in a transforming landscape. Front Health Inform.

[8] Luo Y, Pang PCI, Chang S (2024). Enhancing exploratory learning through exploratory search with the emergence of large language models.

[9] Yang L, Zhang Z, Song Y (2024). Diffusion models: a comprehensive survey of methods and applications. ACM Comput Surv.

[10] Creswell A, White T, Dumoulin V, Arulkumaran K, Sengupta B, Bharath AA (2018). Generative adversarial networks: an overview. IEEE Signal Process Mag.

[11] Sai S, Gaur A, Sai R, Chamola V, Guizani M, Rodrigues J (2024). Generative AI for transformative healthcare: a comprehensive study of emerging models, applications, case studies, and limitations. IEEE Access.

[12] Holley K, Mathur M (2024). LLMs and Generative AI for Healthcare: The Next Frontier.

[13] Arjunan G (2024). AI beyond text: integrating vision, audio, and language for multimodal learning. International Journal of Innovative Science and Research Technology.

[14] Webber G, Reader AJ (2024). Diffusion models for medical image reconstruction. BJR|Artificial Intelligence.

[15] Khang A, Jadhav B, Sayyed M (2024). Role of Cutting-Edge Technologies and Deep Learning Frameworks in the Digital Healthcare Sector AI-Driven Innovations in Digital Healthcare: Emerging Trends, Challenges.

[16] Arets TT, Perugia G, Houben M, Ijsselsteijn WA (2025). The role of generative AI in facilitating social interactions: a scoping review. arXiv.

[17] Aad SS, Hardey M (2025). GAI as a Persona in Education: Enhancing Interaction and Engagement.

[18] Rodríguez-Martínez A, Amezcua-Aguilar T, Cortés-Moreno J, Jiménez-Delgado JJ (2023). Qualitative analysis of conversational chatbots to alleviate loneliness in older adults as a strategy for emotional health. Healthcare (Basel).

[19] Almansour M, Alfhaid FM (2024). Generative artificial intelligence and the personalization of health professional education: a narrative review. Medicine (Baltimore).

[20] Dey AK (2023). ChatGPT in diabetes care: an overview of the evolution and potential of generative artificial intelligence model like ChatGPT in augmenting clinical and patient outcomes in the management of diabetes. International Journal of Diabetes and Technology.

[21] Göktaş M, Bilgehan T (2025). A new era in diabetes management: generative artificial intelligence. Artificial Intelligence Theory and Applications.

[22] Liu T, Pang PCI, Lam CK (2024). Public health education using social learning theory: a systematic scoping review. BMC Public Health.

[23] Chen Q, Wu Y, Li C (2024). Risk management and application of artificial intelligence in elderly patients with chronic diseases and sarcopenia. Journal of Medicine and Health Science.

[24] Atalor SI, Enyejo JO (2025). Mobile health platforms for medication adherence among oncology patients in rural populations. International Journal of Innovative Science and Research Technology (IJISRT).

[25] Vuong QP (2024). The potential for artificial intelligence and machine learning in healthcare: the future of healthcare through smart technologies [Bachelor’s Thesis]. https://www.theseus.fi/handle/10024/866066.

[26] Song X, Liu C, Xu L, Gao B, Lu Z, Zhang Y Affective computing methods for multimodal embodied AI human–computer interaction. Aslib Journal of Information Management.

[27] Lopez-Barreiro J, Garcia-Soidan JL, Alvarez-Sabucedo L, Santos-Gago JM (2024). Artificial intelligence-powered recommender systems for promoting healthy habits and active aging: a systematic review. Appl Sci (Basel).

[28] Rethlefsen ML, Kirtley S, Waffenschmidt S (2021). PRISMA-S: an extension to the PRISMA statement for reporting literature searches in systematic reviews. Syst Rev.

[29] Hong QN Revision of the Mixed Methods Appraisal Tool (MMAT): A Mixed Methods Study.

[30] Satoh M, Inoue J, Ogawa JI (2024). Transforming text to music using artificial intelligence improves the frontal lobe function of normal older adults. Brain Behav.

[31] Pramod D, Vijayakumar Bharathi S, Patil K, Pramod D, Bharathi SV, Patil K Generative artificial intelligence for personalized elderly care through the lens of the hedonic motivation system adoption model.

[32] Xie Y, Li M, Zeng S, Mo J, Diao Y, Liu G (2024). FitPal: reshape daily exercise misconceptions among elders through AI chatbot and community-based services. https://dl.acm.org/doi/10.1145/3678884.3681880.

[33] Yu Y, Shuai Y, Wan R (2024). Exploring the effects of horticultural therapy combined with AIGC on depression in community-dwelling elderly. https://scispace.com/pdf/exploring-the-effects-of-horticultural-therapy-combined-with-3hl5tvbyl3.pdf.

[34] Jin Y, Cai W, Chen L, Zhang Y, Doherty G, Jiang T (2024). Exploring the design of generative AI in supporting music-based reminiscence for older adults. https://dl.acm.org/doi/proceedings/10.1145/3613904.

[35] Yun BH, Kim W, Ko HJ (2024). Development and effectiveness of an AI chatbot-based mobile cognitive screening and customized training application for preventing dementia: older adults living in rural areas of South Korea. Archives of Design Research.

[36] Dragut AC, Lacuesta R, Gallardo J, Buades-Rubio JM (2025). Design of an AI-based language correction system to improve older adults’ interaction with voice assistants. Univ Access Inf Soc.

[37] Khan MA, Din IU, Khan NA, Hassan S, Almogren A Adaptive generative AI for elderly-assisted living environments: a proactive approach.

[38] McCarren L, Kuoppamäki S (2025). Intelligent Health Systems–From Technology to Data and Knowledge.

[39] Tsai WC, Hsieh YC, Lee CF Exploring effectiveness of absorbing health knowledge by the middle-aged and elderly using chatbots.

[40] Chou YH, Lin C, Lee SH, Lee YF, Cheng LC (2024). User-friendly chatbot to mitigate the psychological stress of older adults during the COVID-19 pandemic: development and usability study. JMIR Form Res.

[41] Bennion MR, Hardy GE, Moore RK, Kellett S, Millings A (2020). Usability, acceptability, and effectiveness of web-based conversational agents to facilitate problem solving in older adults: controlled study. J Med Internet Res.

[42] Reed JM, Dodson T, Petrinec A, Hughes J, Miller RD (2025). The HARMONEE project: using genAI images for reminiscence with older adults in long-term care. Geriatr Nurs (Lond).

[43] Bosco C, Shojaei F, Theisz AA (2024). Testing 3 modalities (voice assistant, chatbot, and mobile app) to assist older African American and black adults in seeking information on Alzheimer disease and related dementias: Wizard of Oz usability study. JMIR Form Res.

[44] Naseer F, Addas A, Tahir M, Khan MN, Sattar N (2025). Integrating generative adversarial networks with IoT for adaptive AI-powered personalized elderly care in smart homes. Front Artif Intell.

[45] Kim Y, Kang Y, Kim B, Kim J, Kim GH (2025). Exploring the role of engagement and adherence in chatbot-based cognitive training for older adults: memory function and mental health outcomes. Behav Inf Technol.

[46] Bosco C, Otenen E, Osorio Torres J (2025). Designing a multimodal and culturally relevant Alzheimer disease and related dementia generative artificial intelligence tool for black American informal caregivers: cognitive walk-through usability study. JMIR Aging.

[47] Olszewski R, Watros KM, Brzeziński J (2025). COVID-19 health communication strategies for older adults: chatbots and traditional media. Adv Clin Exp Med.

[48] Liang X, Sun F, Zhang Q (2025). Chatbot-delivered stage of change-tailored web-based intervention to promote physical activity among inactive community-dwelling people aged 65 years or more: protocol for a randomized controlled trial. JMIR Res Protoc.

[49] Miura C, Chen S, Saiki S, Nakamura M, Yasuda K (2022). Assisting personalized healthcare of elderly people: developing a rule-based virtual caregiver system using mobile chatbot. Sensors (Basel).

[50] Wang X, Liang T, Li J (2021). Artificial intelligence-empowered chatbot for effective COVID-19 information delivery to older adults. International Journal of E-Health and Medical Communications.

[51] Thetbanthad P, Sathanarugsawait B, Praneetpolgrang P (2025). Application of generative artificial intelligence models for accurate prescription label identification and information retrieval for the elderly in northern east of Thailand. J Imaging.

[52] Khamaj A (2025). AI-enhanced chatbot for improving healthcare usability and accessibility for older adults. Alexandria Engineering Journal.

[53] Imkome EU, Soonthornchaiya R, Lakanavisid P (2025). Ai-aun chatbot: a pilot study on the effectiveness of an artificial intelligence intervention for mental health among Thai older adults. Nurs Health Sci.

[54] Piau A, Crissey R, Brechemier D, Balardy L, Nourhashemi F (2019). A smartphone chatbot application to optimize monitoring of older patients with cancer. Int J Med Inform.

[55] Lin S, Duan Y, Zhou T, Liu X, Wang J (2025). EHMQA-GPT: a knowledge augmented large language model for personalized elderly health management. Information.

[56] Wang Z, Chen S, Poon J (2025). A hybrid chatbot to promote pneumococcal vaccination among older adults: a randomized clinical trial. JAMA Netw Open.

[57] Wang Y, Li S (2024). Tech vs. tradition: ChatGPT and mindfulness in enhancing older adults’ emotional health. Behav Sci (Basel).

[58] Tricco AC, Lillie E, Zarin W (2018). PRISMA extension for scoping reviews (PRISMA-ScR): checklist and explanation. Ann Intern Med.

[59] Newman DB, Sachs ME, Stone AA, Schwarz N (2020). Nostalgia and well-being in daily life: an ecological validity perspective. J Pers Soc Psychol.

[60] Bodas H (2024). Optimizing healthcare with AI chatbots: addressing challenges and opportunities. SSRN.

[61] Xian X (2024). Unveiling the future: how can GAI transform mental health care?. MHM.

[62] Theil Cabreira A (2019). An investigation of mid-air gesture interaction for older adults [PhD thesis].

[63] Kalyuga S (2008). Managing Cognitive Load in Adaptive Multimedia Learning.

[64] Wach K, Dương DD, Ejdys J (2023). The dark side of generative artificial intelligence: a critical analysis of controversies and risks of ChatGPT. Entrepreneurial Business and Economics Review.

[65] Marzo RR, Klimczuk A (2024). Intergenerational Relations-Contemporary Theories, Studies and Policies.

[66] Ortega-Ochoa E, Sabaté JM, Arguedas M, Conesa J, Daradoumis T, Caballé S (2024). Exploring the utilization and deficiencies of generative artificial intelligence in students’ cognitive and emotional needs: a systematic mini-review. Front Artif Intell.

[67] Wong Y, Neo XS (2025). Smart cities for aging populations: future trends in age-friendly public health policies. Journal of Foresight and Health Governance.

[68] Zhao T (2018). Designing a mobile reading user interface for aging populations. http://rave.ohiolink.edu/etdc/view?acc_num=kent152422790328748.

[69] Espinoza F, Cook D, Da Re M, Fuentes MSC, Butler CR, Calvo RA (2025). Designing AI-powered chatbots for dementia care in Peru: stakeholder engagement and field observations. Interact Comput.

[70] Korobenko D, Nikiforova A, Sharma R (2024). Towards a Privacy and Security-Aware Framework for Ethical AI: Guiding the Development and Assessment of AI Systems. https://dl.acm.org/doi/proceedings/10.1145/3657054.

[71] Hine C, Barnaghi P Ethics and artificial intelligence in the interdisciplinary collaborations of smart care. Sci Technol Human Values.

