# Supplementary material for: The Role of Multimodal Generative AI in Older Adults’ Health Management: Systematic Scoping Review
Source: JMIR AI. 2026 May 29;5:e84695. doi: 10.2196/84695 (PMC13220790; doi:10.2196/84695)
Supplement: Checklist 2 [file ai-v5-e84695-s002.docx]

**Appendix B**

**PRISMA-S Checklist**

| **Section/topic** | **#** | **Checklist item** | **Location(s) Reported** |
| --- | --- | --- | --- |
| **INFORMATION SOURCES AND METHODS** | | | |
| Database name | 1 | Name each individual database searched, stating the platform for each. | Eleven databases were searched: Web of Science, Scopus, PubMed, Medline, CINAHL, Cochrane, ACM Digital Library, IEEE Xplore, ScienceDirect, APA PsycInfo, and Google Scholar. |
| Multi-database searching | 2 | If databases were searched simultaneously on a single platform, state the name of the platform, listing all of the databases searched. | Searches were run individually within each database interface to allow for customized search syntax. No multi-database platforms were used simultaneously. |
| Study registries | 3 | List any study registries searched. | No study registries (e.g., ClinicalTrials.gov) were searched, as the review's objective was to identify finalized and published studies on GenAI's practical application and efficacy, rather than ongoing or unpublished clinical trials. |
| Online resources and browsing | 4 | Describe any online or print source purposefully searched or browsed (e.g., tables of contents, print conference proceedings, web sites), and how this was done. | Google Scholar was purposefully searched to identify non-indexed or difficult-to-locate conference proceedings and relevant grey literature. |
| Citation searching | 5 | Indicate whether cited references or citing references were examined, and describe any methods used for locating cited/citing references (e.g., browsing reference lists, using a citation index, setting up email alerts for references citing included studies). | Reference lists of all included studies (n=28) were manually screened, and forward citation tracking was performed using Web of Science and Scopus. |
| Contacts | 6 | Indicate whether additional studies or data were sought by contacting authors, experts, manufacturers, or others. | Authors or experts were not contacted. Due to the scope and time constraints of the review, the study relied primarily on multi-database searches and citation tracking to ensure the objectivity and replicability of the data extraction process. |
| Other methods | 7 | Describe any additional information sources or search methods used. | Tables of contents for the three most relevant journals and major computer science conferences from 2023 to 2025 were manually screened to ensure comprehensiveness. |
| **SEARCH STRATEGIES** | | | |
| Full search strategies | 8 | Include the search strategies for each database and information source, copied and pasted exactly as run. | The full Boolean search strategy for each of the 11 databases is provided verbatim in Table 1. |
| Limits and restrictions | 9 | Specify that no limits were used, or describe any limits or restrictions applied to a search (e.g., date or time period, language, study design) and provide justification for their use. | Searches were restricted by language (English) and publication type (Research Articles and Conference Proceedings). |
| Search filters | 10 | Indicate whether published search filters were used (as originally designed or modified), and if so, cite the filter(s) used. | No published methodological filters (e.g., RCT filters) were used. The search strategy was designed to maximize sensitivity to capture a broad spectrum of study designs, with all limitations achieved through the precise combination of subject headings and Boolean operators to accurately align with the two core concepts: elderly health and GenAI. |
| Prior work | 11 | Indicate when search strategies from other literature reviews were adapted or reused for a substantive part or all of the search, citing the previous review(s). | Search strategies were developed *de novo* by the review team; no previous literature review strategies were adapted or reused. |
| Updates | 12 | Report the methods used to update the search(es) (e.g., rerunning searches, email alerts). | An update search was conducted one month after the initial search by re-running the exact search strings in all 11 databases to capture the most recent publications. |
| Dates of searches | 13 | For each search strategy, provide the date when the last search occurred. | The final, most recent search was conducted on July 28, 2025. |
| **PEER REVIEW** | | | |
| Peer review | 14 | Describe any search peer review process. | The search strategy was internally peer-reviewed by two independent authors (T.L. and Y.L.) who checked the syntax, logic, and field codes for accuracy and completeness before running the final searches. |
| **MANAGING RECORDS** | | | |
| Total Records | 15 | Document the total number of records identified from each database and other information sources. | A total of 69,074 records were initially identified across all 11 databases. The specific number of records per database is detailed in the PRISMA Flow Diagram (Figure 1). |
| Deduplication | 16 | Describe the processes and any software used to deduplicate records from multiple database searches and other information sources. | All retrieved records were exported to EndNote software for automated deduplication, followed by a manual check by one author (T.L.) to ensure the complete removal of all remaining duplicates. |
|  |  |  |  |
| PRISMA-S: An Extension to the PRISMA Statement for Reporting Literature Searches in Systematic Reviews | | |  |
| Rethlefsen ML, Kirtley S, Waffenschmidt S, Ayala AP, Moher D, Page MJ, Koffel JB, PRISMA-S Group. | | |  |
| Last updated February 27, 2020. | |  |  |
